# Hof1 and Rvs167 Have Redundant Roles in Actomyosin Ring Function during Cytokinesis in Budding Yeast

**DOI:** 10.1371/journal.pone.0057846

**Published:** 2013-02-28

**Authors:** Pedro Junior Nkosi, Bianca-Sabrina Targosz, Karim Labib, Alberto Sanchez-Diaz

**Affiliations:** Paterson Institute for Cancer Research, University of Manchester, Manchester, United Kingdom; Karolinska Institutet, Sweden

## Abstract

The Hof1 protein (Homologue of Fifteen) regulates formation of the primary septum during cytokinesis in the budding yeast *Saccharomyces cerevisiae*, whereas the orthologous Cdc15 protein in fission yeast regulates the actomyosin ring by using its F-BAR domain to recruit actin nucleators to the cleavage site. Here we show that budding yeast Hof1 also contributes to actin ring assembly in parallel with the Rvs167 protein. Simultaneous deletion of the *HOF1* and *RVS167* genes is lethal, and cells fail to assemble the actomyosin ring as they progress through mitosis. Although Hof1 and Rvs167 are not orthologues, they both share an analogous structure, with an F-BAR or BAR domain at the amino terminus, capable of inducing membrane curvature, and SH3 domains at the carboxyl terminus that bind to specific proline-rich targets. The SH3 domain of Rvs167 becomes essential for assembly of the actomyosin ring in cells lacking Hof1, suggesting that it helps to recruit a regulator of the actin cytoskeleton. This new function of Rvs167 appears to be independent of its known role as a regulator of the Arp2/3 actin nucleator, as actin ring assembly is not abolished by the simultaneous inactivation of Hof1 and Arp2/3. Instead we find that recruitment to the bud-neck of the Iqg1 actin regulator is defective in cells lacking Hof1 and Rvs167, though future studies will be needed to determine if this reflects a direct interaction between these factors. The redundant role of Hof1 in actin ring assembly suggests that the mechanism of actin ring assembly has been conserved to a greater extent across evolution than anticipated previously.

## Introduction

When animal cells and fungi pass through mitosis, a contractile ring of actin, type II myosin and many other factors assembles under the plasma membrane between the two separated nuclei, and plays a central role in dividing the cytoplasm during cytokinesis [1]. The presence of a cell wall outside the plasma membrane in fungi means that membrane ingression during cytokinesis is coupled tightly to the synthesis of primary and secondary septa at the cleavage site, and the subsequent digestion of the primary septum completes cell division in yeast cells [2]. There is an intimate connection between the actomyosin ring and septum formation in yeasts, such that the actin ring guides the efficient formation of a primary septum, and septum formation stabilises the contracting ring [3], [4], [5], [6].

In both budding and fission yeasts, assembly of the actomyosin ring requires formins and the IQGAP protein, which nucleate and/or bundle actin filaments [7], [8], [9]. In the fission yeast *Schizosaccharomyces pombe*, formin is recruited to the middle of the cell during mitosis, in part by the Cdc15 protein [10], [11], [12], which is a key regulator of the assembly and stability of the actomyosin ring [12], [13], [14]. The amino terminus of Cdc15 comprises an F-BAR domain (F-BAR  =  ‘FCH and BAR’, where FCH  =  Fes/CIP4 Homology and BAR  =  Bin-Amphiphysin-Rvs), which is a curved protein module that binds to membranes and induces tubulation [15], [16], and also binds directly to formin and other actin regulators [10]. The carboxyl terminus of Cdc15 contains an SH3 domain that plays a redundant role with the SH3 of the related F-BAR protein Imp2 [17], in recruiting factors that contribute by an unknown mechanism to stability of the actomyosin ring during contraction [18].

The orthologue of Cdc15 in the budding yeast *Saccharomyces cerevisiae* is known as Hof1 (Homologue of Fifteen) and also plays an important role during cytokinesis [19], [20], [21]. Hof1 is dispensable for assembly of the actomyosin ring, however [20]–[21], and until now was thought to play a rather different role to fission yeast Cdc15. Previous studies showed that Hof1 acts in parallel with another factor Cyk3, to stimulate formation of the primary septum by Chitin synthase II [21], [22], [23]. Both Hof1 and Cyk3 interact via their SH3 domains with Inn1 (required for Ingression), which is essential for formation of the primary septum [23], and is a positive regulator of chitin synthase II [26].

Here we identify a novel requirement for budding yeast Hof1 in assembly of the actomyosin ring, in addition to its other roles in septation. Our data indicate that this role of Hof1 is redundant with the SH3 domain of Rvs167, which was a founder member of the BAR domain family. These findings suggest that the mechanism of actin ring assembly in budding yeast involves considerable redundancy between the factors that recruit actin nucleators, but will prove to be fundamentally similar to actin ring assembly in fission yeast.

## Results

### The SH3 domain of Rvs167 becomes essential in the absence of Hof1

Hof1 is essential for septum formation at 37°C but *hof1Δ* cells are viable at lower temperatures [19], [20], [21], suggesting redundancy with other factors during cytokinesis. Indeed, previous work showed that the SH3 domain of Cyk3 becomes essential in the absence of Hof1 [22], [24], and further studies showed that Cyk3 stimulates septum formation during cytokinesis 23,26,27.

**Figure 1:**
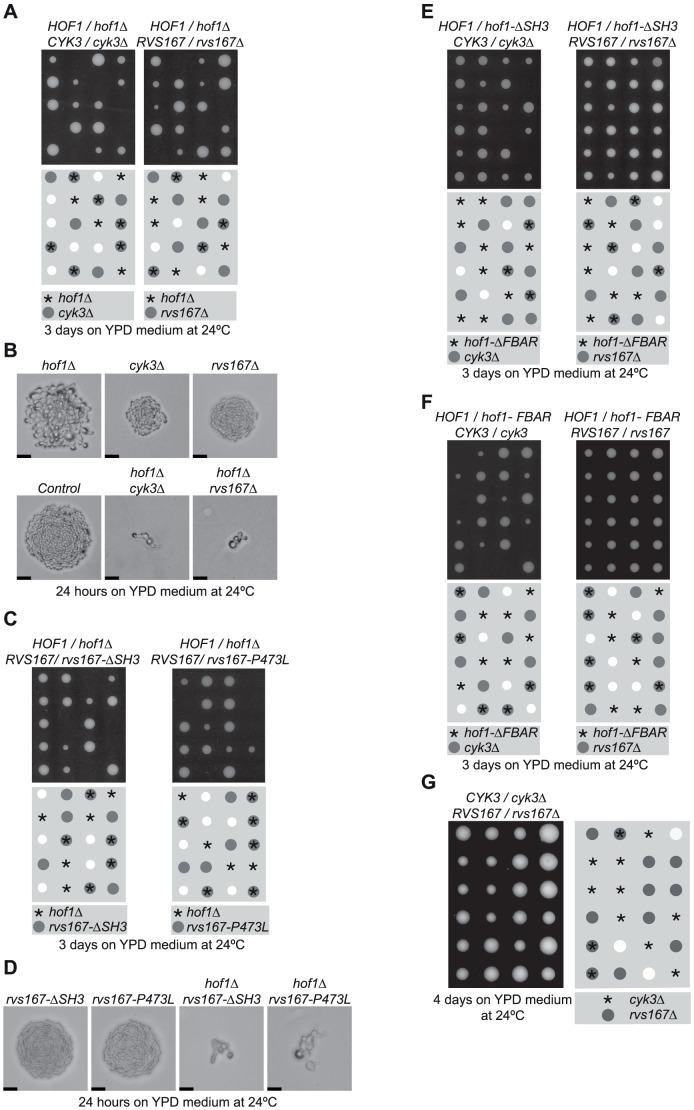
The SH3 domain of Rvs167 becomes essential for cell proliferation in the absence of Hof1. (**A**) Tetrad analysis of diploid yeast cells lacking one copy of *HOF1* and one copy of either *RVS167* or *CYK3*. (**B**) Spores of the indicated genotypes were grown for 24 hours on YPD plates at 24°C. The scale bars indicate 20 µm. (**C-D**) Similar experiments illustrating that the SH3 domain of Rvs167 is essential in cells lacking Hof1. (**E**) The SH3 domain of Hof1 is essential in the absence of Cyk3 but dispensable in the absence of Rvs167. (**F**) The same is true for the F-BAR domain of Hof1. (**G**) Rvs167 does not become essential in the absence of Cyk3.

In addition to Hof1, two other non-essential budding yeast proteins combine carboxy terminal SH3 domains with an F-BAR (Bzz1) or BAR (Rvs167) domain at the amino terminus. The Bzz1 protein is a component of cortical actin patches and interacts via its SH3 domain with regulators of the Arp2/3 actin nucleator [28]. We generated budding yeast cells that lacked both Hof1 and Bzz1, but did not observe a synthetic growth defect (BT, ASD and KL, unpublished data). Like Bzz1, the Rvs167 protein is a component of actin patches that interacts with Arp2/3 [29], [30], [31], [32]. Very recent work has shown by bimolecular fluorescence that Rvs167 also co-localises at the bud-neck with other cytokinesis factors such as Inn1, Cyk3 and Iqg1 (Mike Cundell and Clive Price, personal communication). We sporulated diploid cells lacking one copy of *HOF1* as well as one copy of *RVS167*, and found by tetrad analysis of the meiotic progeny that the combination of *hof1Δ* with *rvs167Δ* was lethal (Figure 1A). Moreover, *hof1Δ rvs167Δ* cells had a rather similar phenotype to *hof1Δ cyk3Δ* (Figure 1B), suggestive of a defect in some aspect of cell cycle progression. It thus seems that Rvs167 becomes essential for cell proliferation in the absence of Hof1. In contrast, Rvs167 is not essential in cells lacking Cyk3 (Figure 1G).

The BAR domain of Rvs167 is essential for the previously described functions of Rvs167, and together with the related BAR domain of Rvs161 forms a heterodimeric complex that is able to induce membrane curvature and tubule formation *in vitro* [33], [34], [35]. Conversely, the SH3 domain of Rvs167 is dispensable for most known of the previously identified roles of Rvs167 [33], though it is known to interact with regulators of Arp2/3 (Figure S1). We found that *hof1Δ* was synthetic lethal both with *rvs167-ΔSH3* and also with the *rvs167-P473L* allele (Figure 1C, D) that specifically kills the function of the SH3 domain [33]. These findings suggested that the SH3 domain of Rvs167 might play a redundant role with Hof1 during cell division in budding yeast. To confirm the specificity of the observed genetic interactions, we also used tetrad analysis to combine *hof1Δ* with deletions of each of the genes encoding the other 20 non-essential SH3 proteins in budding yeast (in addition to Hof1, Rvs167, Cyk3 and Bzz1; we could not test Bem1 as it is essential in the W303 yeast strain with which we work). The only other synthetic lethal combination was *hof1Δ sho1Δ* (Figure S2A, and BT, ASD and KL, unpublished data), probably reflecting an essential role for the Sho1 branch of the Hog1 MAP kinase pathway in the absence of Hof1, as *hog1Δ* and *pbs2Δ* (Pbs2 is the MAP kinase kinase for Hog1) were also synthetic lethal with *hof1Δ* (Figure S2B, C). In contrast, *rvs167Δ* was not synthetic lethal with *sho1Δ* (Figure S2D).

We also tested which part of the Hof1 protein was most important in the absence of Rvs167. Whereas removal of either the F-BAR or SH3 domains of Hof1 was lethal in cells lacking Cyk3, neither *hof1-ΔSH3* nor *hof1-ΔFBAR* were lethal in combination with *rvs167Δ* or *rvs167-ΔSH3* (Figure 1E, F; Figure S3A, B). It thus appears that the F-BAR and SH3 domains of Hof1 play a redundant role that becomes essential in the absence of Rvs167. Alternatively, viability in the absence of Rvs167 might be dependent upon the central region of Hof1, which targets the protein to the medial ring during anaphase [36].

### Hof1 and the SH3 domain of Rvs167 both contribute to assembly of the actomyosin ring

**Figure 2:**
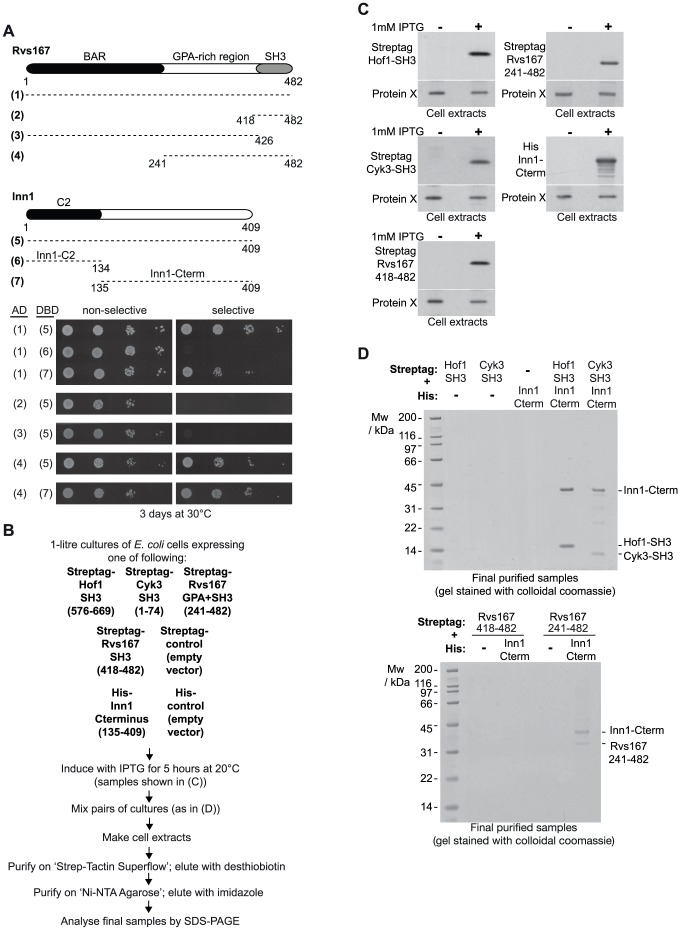
Physical interactions between Rvs167 and Inn1. (**A**) Truncated alleles of Rvs167 and Inn1 were used to show that the region of Rvs167 after the BAR domain (Rvs167 241-482) can interact in a 2-hybrid assay with the Proline-rich region of Inn1 after its C2 domain (Inn1 135-409). (**B**) Scheme explaining how the indicated protein fragments were expressed in cultures of *E. coli* cells, and then mixed to allow the purification of protein complexes. After induction with IPTG, pairs of cultures were mixed as indicated in (D) below, and used to purify protein complexes between the induced proteins, via Strep-Tactin Superflow and Ni-NTA agarose resins (see Methods). (**C**) Immunoblots showing induction of the various protein fragments listed in (B). The tagged proteins were detected with anti-Streptag or anti-His antibodies. In each case, a non-specific band corresponding to an unknown *E. coli* protein is included to provide a loading control. (**D**) Inn1 135-409 can interact directly to form a stable complex with the SH3 domains of Hof1 and Cyk3, as well as with Rvs167 241-482. Pairs of *E. coli* cell cultures expressing the indicated protein fragments were mixed and used to purify putative protein complexes as shown in (B). The final purified fractions were analysed by SDS-PAGE and the gels were stained with colloidal Coomassie blue.

Hof1, Cyk3 and Iqg1 all interact with Inn1 [23], [24], [25], and we used the 2-hybrid assay to show that the same was true for Rvs167 (Figure 2A). The interaction was specific, as a range of other proteins with SH3 domains were unable to associate with Inn1 in the same assay (Figure S4A; [24]). The interaction required the function of the SH3 domain of Rvs167 (Figure S4B) but also involved the region rich in Glycine-Proline-Alanine that separates the BAR and SH3 domains of Rvs167 (Figure 2A). An equivalent fragment of Rvs167 co-purified specifically with the Proline-rich region of Inn1 from an extract of *E. coli* cells, whereas the SH3 domain alone did not (Figure 2B-D). This shows that the interaction of Rvs167 and Inn1 is direct and also explains why it was not detected in previous systematic studies of the targets of yeast SH3 proteins including Rvs167, as these studies focussed on the minimal SH3 domains [37], [38].

**Figure 3:**
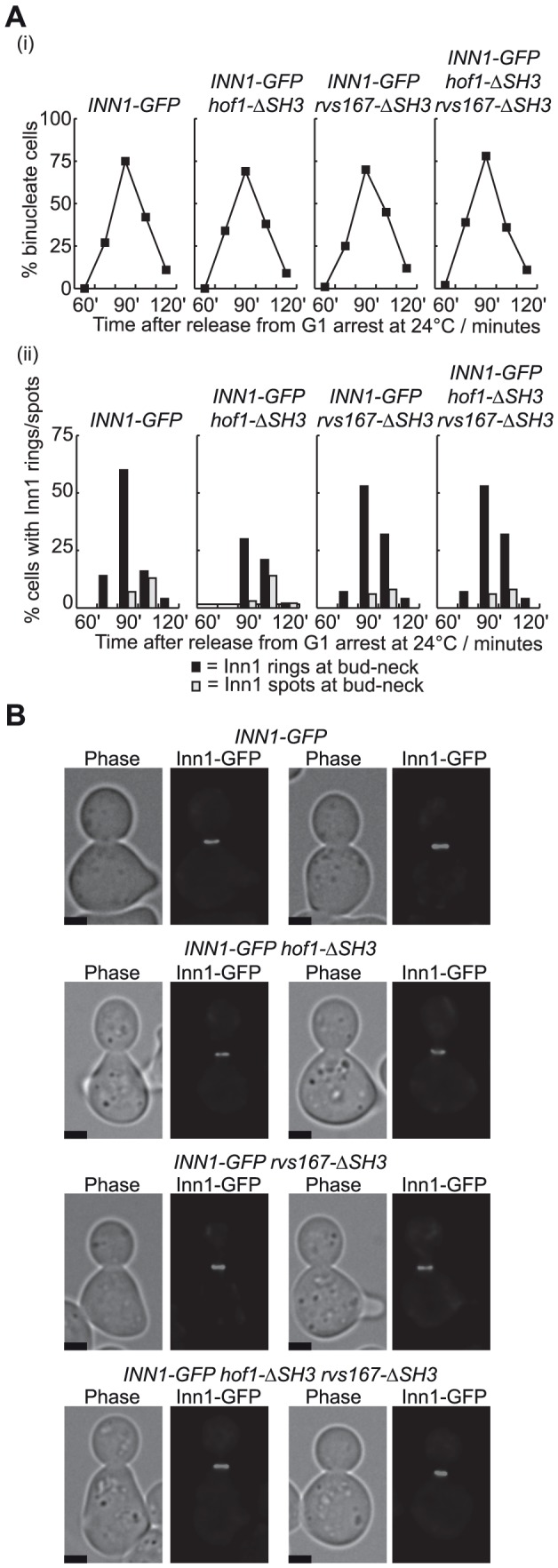
Inn1 can still be recruited to the bud-neck in the absence of the SH3 domains of Rvs167 and Hof1. (**A**) The indicated strains were released from G1-arrest at 24°C and allowed to progress through the cell cycle. The proportion of binucleate cells was monitored in parallel with recruitment of Inn1 to the bud-neck. (**B**) Examples of cells with Inn1-GFP rings at the bud-neck are shown for the 90’ time-point in (A). The scale-bars indicate 2 µm.

**Figure 4:**
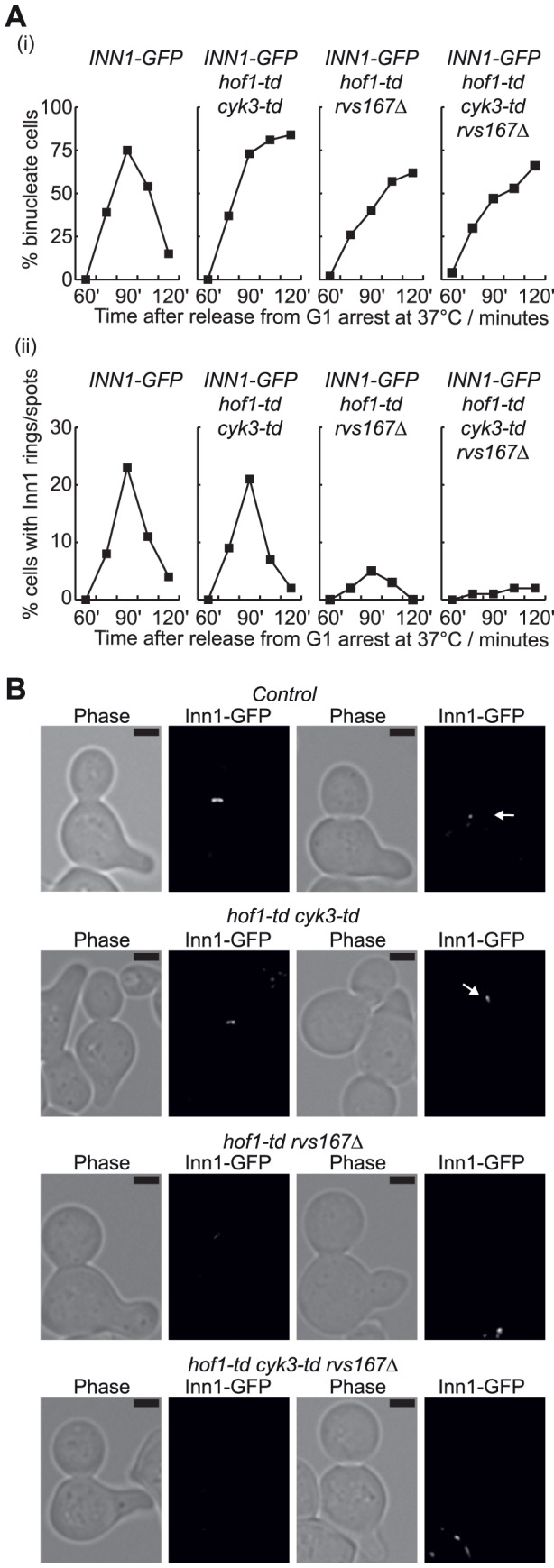
Recruitment of Inn1 to the bud-neck is defective in the complete absence of Hof1 and Rvs167. (**A**) The indicated strains were synchronised in G1 phase at 24°C, before expression of *GAL-UBR1* (Ubr1 is the E3 ligase for N-end rule pathway that mediates ubiquitylation of the heat inducible degron) and degradation of Hof1-td and Cyk3-td at 37°C. Cells were then released from G1 arrest and samples taken at the indicated times to determine the proportion of bi-nucleate cells (i) and the percentage of cells with rings or spots of Inn1 at the bud-neck (ii), as cells completed the cell cycle. (**B**) Images from the experiment described in (A). The Inn1-GFP rings in *hof1-td cyk3-td* were frequently less bright than those observed in control cells. The scale bars correspond to 2 µm.

Inn1 must be recruited to the bud-neck at the end of mitosis to activate chitin synthase II, and recruitment of Inn1 is jointly dependent upon Hof1 and the integrity of the actomyosin ring [23], [25]. It seemed possible that Rvs167 might not have a direct role in recruitment of Inn1, as the *hof1-ΔSH3* allele is not synthetic lethal with *rvs167Δ* (Figure 1E), and we found that Inn1 was still recruited to the budneck at the end of mitosis in *hof1-ΔSH3 rvs167-ΔSH3* cells that are viable at 24°C (Figure 3). Surprisingly, however, we found that recruitment of Inn1 to the bud-neck was greatly defective in the complete absence of Rvs167 and Hof1. Whereas Inn1 appeared at the bud-neck during late mitosis in control cells, or following the rapid depletion of Hof1 and Cyk3 in cells with both proteins fused to the heat inducible degron [39], [40], recruitment of Inn1 was severely compromised in *hof1-td rvs167Δ* cells and abolished in *hof1-td cyk3-td rvs167Δ* (Figure 4A-B; td  =  temperature sensitive degron), despite equivalent defects in cell division in all three strains that lacked Hof1 at 37°C (Figure 4A (i)).

**Figure 5:**
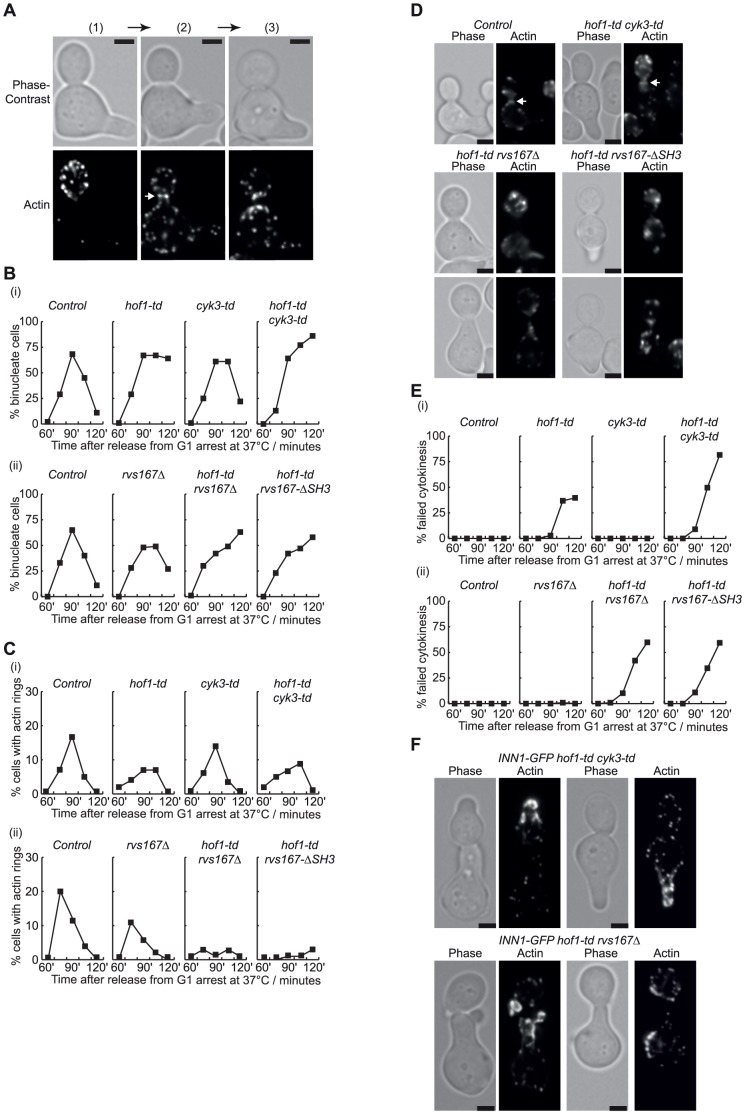
Assembly of the actin ring is defective in cells lacking both Hof1 and Rvs167. A similar range of strains to those described above for Figure 4 were grown as before, and assembly of the actin ring was monitored in time-course experiments by staining fixed cells with rhodamine phalloidin. (**A**) In control cells, actin patches accumulate in the new bud until mitosis (1), before depolarising throughout the cell during anaphase when the actin ring assembles (2; marked by arrow). Contraction of the actomyosin ring is associated with the accumulation of actin patches on either side of the bud-neck (3). The scale bars correspond to 2 µm. (**B**) The proportion of bi-nucleate cells was determined throughout two sets of time-course experiments involving inactivation of Hof1-td and Cyk3-td (i), or combination of Hof1-td with *rvs167* or *rvs167-SH3* (ii). Samples from the same time-course experiments were stained with rhodamine phalloidin and used to determine the proportion of cells with actin rings (**C**-**D**) and the percentage of cells that failed to complete cytokinesis (**E**). Images of *hof1-td cyk3-td* and *hof1-td rvs167* cells that failed to complete cytokinesis are shown in panel (**F**), in which the scale bars correspond to 2 µm.

A previous study showed that recruitment of Inn1 to the cleavage site is blocked by treatment of *hof1Δ* cells with the drug Latrunculin A that depolymerises actin and prevents assembly of the actomyosin ring [23]. It thus seemed possible that a defect in assembling the actin ring could contribute to the defective recruitment of Inn1 in cells lacking Rvs167 and Hof1. In similar experiments to those described above, we used rhodamine-phalloidin staining to monitor the actin cytoskeleton as G1-phase cells passed synchronously through a complete cell cycle at 37°C. In control cells, assembly of the actin ring during mitosis was associated with delocalisation of actin throughout the whole cell, whereas disassembly of the actin ring was followed by the accumulation of actin patches at either side of the bud-neck (Figure 5A).

Consistent with previous studies of *hof1Δ* cells [20]–[21], we observed actin rings when *hof1-td* or *hof1-td cyk3-td* cells completed mitosis at 37°C, despite the fact that 40% of *hof1-td* cells and 80% of *hof1-td cyk3-td* cells failed to complete cell division in the first cell cycle (Figure 5B-F). Nevertheless, the proportion of cells forming actin rings was reduced in both strains compared to the control (Figure 5C (i)). Similarly, actin rings were observed in *rvs167Δ* cells completing the cell cycle at 37°C, but at a reduced frequency (Figure 5C (ii); Figure S5 shows that *rvs167Δ* cells are able to proliferate at 37°C). Most strikingly, the majority of *hof1-td rvs167Δ* cells did not assemble an actin ring (Figure 5C (ii) and 5D), although these cells still exited mitosis and formed actin patches at the bud-neck during their failed attempt to complete cytokinesis, and they subsequently repolarised the actin cytoskeleton to the site of new buds (Figure 5E-F). Considered together, these data indicate that Hof1 and Rvs167 share a redundant role in assembly of the actomyosin ring during cytokinesis in budding yeast.

**Figure 6:**
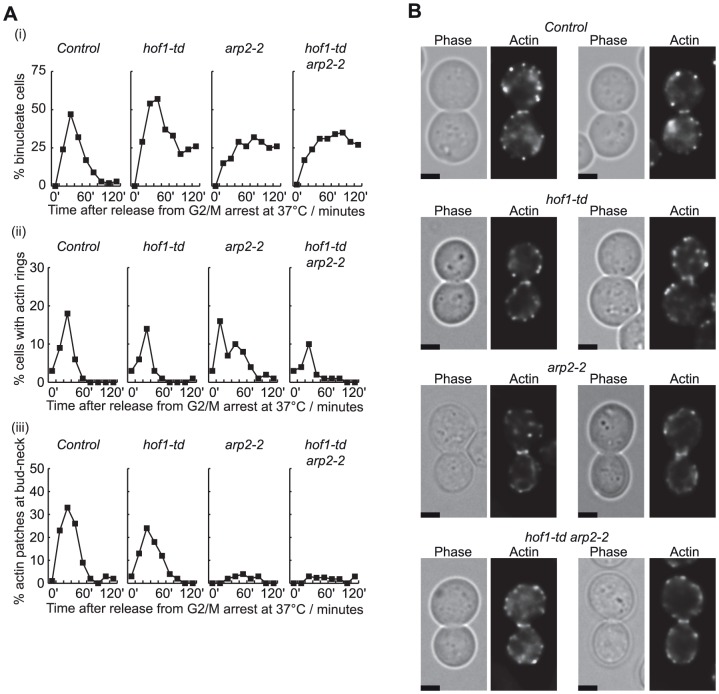
Actin ring assembly still occurs following inactivation of Arp2/3. (**A**) The indicated strains were arrested in G2-M phase with nocodazole at 24°C, before expression of *GAL-UBR1* and incubation at 37°C for 60’ to inactivate Hof1-td and Arp2-2. Cells were then released at 37°C into fresh medium lacking nocodazole, and processed as described above for Figure 5. (**B**) Images of cells with actin rings (marked by arrows), from the 30-minute timepoint in the experiment in (A). The scale bars correspond to 2 µm.

We found that assembly of the actin ring was also abolished in *hof1-td rvs167-ΔSH3* cells (Figure 5C, D), consistent with the fact that the SH3 domain of Rvs167 becomes essential in the absence of Hof1, and suggesting that the SH3 domain of Rvs167 might help to recruit some factor that contributes to assembly of the actomyosin ring. Previous work indicated that the SH3 domain of Rvs167 interacts with regulators of the Arp2/3 complex (Figure S1), though Arp2/3 generates branched actin fibres that are not thought to contribute directly to assembly of the actin ring, in contrast to the structures generated by formin and IQGAP [41]. To test directly whether Arp2/3 played a redundant role with Hof1 in actomyosin ring assembly, we performed similar experiments to those above, with control, *hof1-td*, the temperature sensitive *arp2-2* allele and *hof1-td arp2-2* cells. As Arp2/3 is required for the assembly of actin patches and thus for polarised growth, so that bud assembly is blocked at 37°C in *arp2-2* cells (data not shown), we synchronised cells after bud formation in G2-M phase by addition of nocodazole. The cultures were then shifted to 37°C to inactivate Arp2-2 and deplete Hof1-td, before release into fresh medium lacking nocodazole. As shown in Figure 6A (i), inactivation of either Hof1 or Arp2 at 37°C blocked the completion of cell division. However, actin rings formed in all four strains (Figure 6A (ii) and 6B; the frequency of cells with actin rings was somewhat lower in *hof1-td arp2-2*), whereas the subsequent formation of actin patches at the bud-neck was blocked upon inactivation of Arp2 (Figure 6A (iii)). These data indicated that Arp2/3 was not essential for actin ring formation in the absence of Hof1, suggesting that another partner of the SH3 domain of Rvs167 becomes essential for actin ring formation in the absence of Hof1.

**Figure 7:**
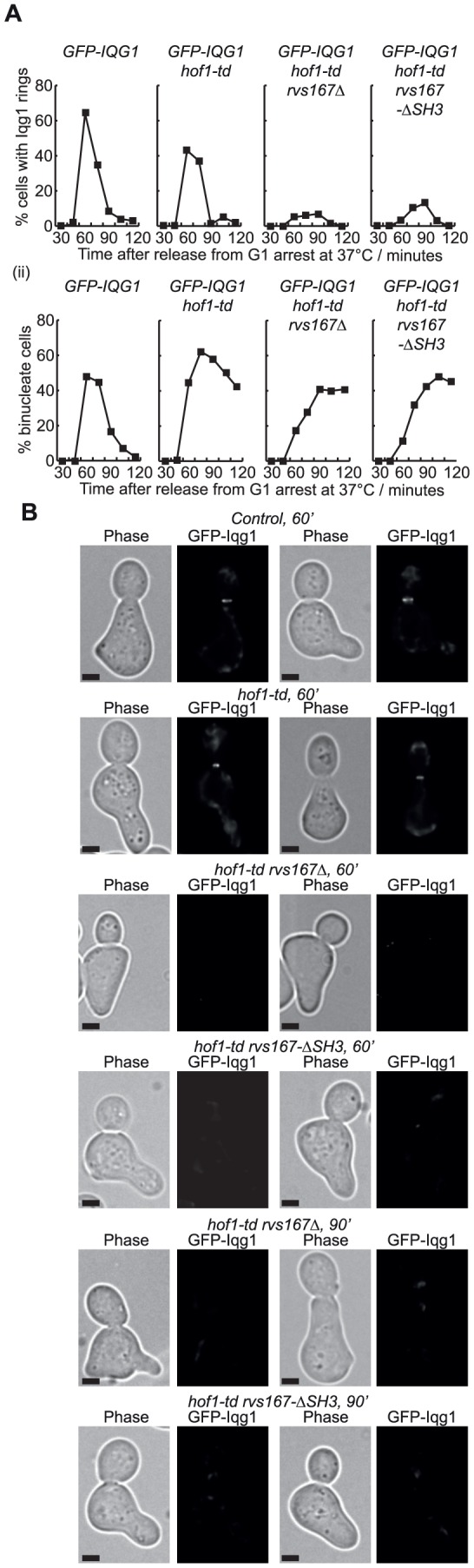
Recruitment of Iqg1 to the bud-neck is defective in cells lacking Hof1 and Rvs167. (**A**) Cells were processed as described above for Figure 4, so that the recruitment of GFP-Iqg1 to the bud-neck (i) could be monitored as cells passed through mitosis (ii). (**B**) Images from the experiment described in (A). The scale bars correspond to 2 µm.

Finally, we examined the recruitment of Iqg1 to the bud-neck in cells lacking Hof1 and Rvs167, in similar experiments to those described above. Whereas Iqg1 was found at the bud-neck during mitosis in control and *hof1-td* cells, recruitment of Iqg1 was very defective, though not completely abolished, in *hof1-td rvs167Δ* and *hof1-td rvs167-ΔSH3* (Figure 7).

## Discussion

Our data identify a novel role for Hof1 in assembly of the actomyosin ring in *Saccharomyces cerevisiae*, in addition to its known role in promoting formation of the primary septum. Consistent with this finding, a recent study of the very similar Hof1 orthologue in the mycelial fungus *Ashbya gossypii* showed that it is essential for assembly of the actomyosin ring [42]. Whereas the SH3 domain of Rvs167 is dispensable for many aspects of Rvs167 function [33], our findings indicate that the SH3 domain of Rvs167 contributes to assembly of the actomyosin ring in parallel to Hof1. Interestingly, recent data indicate that Rvs167 is recruited to the bud-neck in a similar fashion to known regulators of cytokinesis such as Iqg1 and Inn1 (Mike Cundell and Clive Price, personal communication), consistent with our findings. Moreover, we note that a previous study found that Rvs167 and Hof1 were both required for assembly of a ring-like structure in cells upon over-expression of actin [43].

It will be interesting in future studies to determine whether the F-BAR and SH3 domains of Hof1 act redundantly with Rvs167 to recruit actin regulators such as the formins, Iqg1 or other factors to the bud-neck, analogous to the role of the F-BAR domain of fission yeast Cdc15 in recruiting the formin Cdc12 to the middle of the cell during mitosis. We found that recruitment of Iqg1 to the budneck is defective in cells lacking Hof1 and Rvs167, though it remains to be determined whether this defect reflects a direct interaction between these factors, or whether Hof1 and Rvs167 act in other ways to influence the recruitment of Iqg1 indirectly. Like the SH3 domains of Hof1 and Cyk3, the SH3 domain of Rvs167 also interacts with Inn1 (Figure 2). The functional implications remain to be explored, but our data suggest that Rvs167 might not have a direct role in the recruitment of Inn1 to the budneck (Figure 3).

The role of budding yeast Hof1 in assembly of the actomyosin ring shows greater redundancy with other factors than is the case in fission yeast, perhaps reflecting the greater diversity of regulatory factors following an ancient duplication of the budding yeast genome. Nevertheless, it now appears that the basic principles of action of budding yeast Hof1 and fission yeast Cdc15 are likely to be much more similar than anticipated previously, with both proteins contributing to assembly of the actomyosin ring as well as to the stability of the contracting ring and/or septum formation. The SH3 domains of Hof1 and Cyk3 contribute jointly to cytokinesis and septum formation in ways that remain to be characterised in molecular detail, though both interact with Inn1 that activates septum formation by chitin synthase II. Similarly, the SH3 domains of fission yeast Cdc15 and the related F-BAR protein Imp2 play a redundant role in recruiting factors to the cleavage site during cytokinesis, including the Fic1 orthologue of Inn1 as well as at least one other factor [18].

A conserved role for fungal Cdc15/Hof1 proteins in assembly of the actomyosin ring raises the possibility that F-BAR proteins might also regulate the actin cytoskeleton during cytokinesis in animal cells too. This remains to be established in the future, but redundancy between such factors might have hidden a role thus far.

## Methods

### Construction and growth of yeast strains

The strains used in this study are listed in Table SI. Strains were generated and grown as described previously [44], [45]. Yeast cells were grown in rich medium containing 1% yeast extract, 2% peptone and 2% sugar (glucose, raffinose, or galactose) as the only carbon source. We arrested cells in the G1 phase of the cell cycle by addition of alpha factor mating pheromone to the medium at a final concentration of 7.5 µg per ml, and in G2-M phase by addition of nocodazole to 2 µg per ml.

### Microscopy

Pictures of cells and colonies on agar plates were taken after 24 hours with a Nikon CoolPix 995 camera attached to a Nikon Eclipse E400 microscope.

Phase contrast and fluorescence microscopy of cells grown in liquid culture was performed with a Zeiss Axiovert 200 M microscope and a Cool Snap HQ camera (Photometrics) as described previously (Sanchez-Diaz et al, 2008). We analysed Z-stacks with 0.2 µm sections to observe the localisation of Inn1-GFP, GFP-Iqg1 and actin. The microscopy data were deconvolved using Huygens (SVI) as reported previously (Sanchez-Diaz et al, 2008).

For experiments involving Inn1-GFP and GFP-Iqg1, we fixed cells with 8% formaldehyde for 10 minutes, before washing once with PBS. To visualise the actin cytoskeleton, cells were fixed with 3.7% formaldehyde and 0.1% Triton X-100 for 10 minutes. We washed the samples once with PBS, and then incubated for 60 minutes in 3.7% formaldehyde diluted in PBS. Subsequently cells were stained with 0.05 U per µl of rhodamine-phalloidin (Invitrogen) for 90’ before further washing and viewing.

We determined the proportion of binucleate cells by examining ethanol fixed samples that had been treated sequentially with RNase A and pepsin before staining with propidium iodide.

For all experiments involving quantification of microscopy data, we examined at least 100 cells for each sample.

### Yeast Two-hybrid analysis

Two-hybrid analysis was performed using the vectors pGADT7 and pGBKT7 (Clontech). Cells were grown for 3 days at 30°C on Synthetic Complete medium lacking leucine and tryptophan (non-selective), or equivalent medium that also lacked histidine (selective).

### Expression and purification of recombinant proteins in E.coli

To isolate recombinant protein complexes from extracts of *E.coli* cells, we followed the scheme illustrated in Figure 2B. The various protein fragments were expressed individually as ‘3-Streptag’ or ‘6His-tag’ fusions, using plasmids that were based on the ‘pET’ series (Novagen). Cells containing each of the fusions were grown at 37°C initially, and then expression of the recombinant protein fragments was induced with 1 mM IPTG in 1-litre cultures for 5 hours at 20°C (expression was confirmed as shown in Figure 2C, using ‘Penta-His’ antibody (Qiagen; 34660) and ‘StrepMAB-Classic monoclonal antibody’ (2-1507-001, Fisher)). Subsequently, pairs of cultures were mixed as indicated in Figure 2D, so that each cell extract would contain two recombinant proteins, or one recombinant protein in the case of mixtures involving one culture with an empty vector. The Streptag-fusions were then isolated from the cell extracts on 1 ml of Strep-Tactin Superflow (2-1206-025, IBA GmbH), before elution with 2.5 mM d-Desthiobiotin (D1411, Sigma). The eluted material was then diluted and incubated with 1 ml of Ni-NTA Agarose (30230, Qiagen), and bound protein complexes eluted with sequential 0.5 ml aliquots of buffer containing 250 mM Imidazole. Following the addition of 3X Laemmli buffer to the eluted samples, 25 µl of each purified sample was resolved by SDS-PAGE, as shown in Figure 2D.

## Supporting Information

Figure S1 **Interactions between yeast SH3 proteins and regulators of the actin cytoskeleton.** SH3 proteins are shown in red, and each black line represents a physical interaction, based on published data summarised in ‘BIOGRID’ (http://thebiogrid.org/).
(PDF)

Figure S2 **The Sho1-Hog1 MAP kinase pathway becomes essential in the absence of Hof1.** (**A**) Tetrad analysis of diploid yeast cells lacking one copy of *HOF1* and one copy of *SHO1*. Spores of the indicated genotypes were grown for 24 hours on YPD plates at 24°C. The scale bars indicate 20 µm. (**B**) Tetrad analysis of diploid yeast cells lacking one copy of *HOF1* and one copy of either *HOG1* or *PBS2*. (**C**) Spores of the indicated genotypes were grown for 24 hours on YPD plates at 24°C. The scale bars indicate 20 µm. (**D**) Tetrad analysis of diploid yeast cells lacking one copy of *SHO1* and one copy of *RVS167*.
(PDF)

Figure S3 **The** ***rvs167-*Δ*SH3*** **allele is not synthetic lethal with either** ***hof1-*Δ*FBAR*** **or** ***hof1-*Δ*SH3***. (**A**) Tetrad analysis of diploid yeast cells with one copy of *hof1-ΔFBAR* and one copy of *rvs167-ΔSH3*. (**B**) Tetrad analysis of diploid yeast cells with one copy of *hof1-ΔSH3* and one copy of *rvs167-ΔSH3*.
(PDF)

Figure S4 **Interaction of the SH3 domain of Rvs167 with Inn1 is specific.** (**A**) In contrast to Rvs167, the SH3 proteins Abp1 and Lsb3 do not interact with Inn1 in the two-hybrid assay. (**B**) The two-hybrid interaction of Rvs167 with Inn1 is blocked by the Rvs167-P473L mutation in the SH3 domain.
(PDF)

Figure S5 **In contrast to** ***hof1*Δ**** **, the** ***rvs167*Δ**** **strain grows well at 37**°**C.**Cells were grown for two days on YPD medium at 24°C or 37°C as indicated.
(PDF)

Table S1 **Yeast strains used in this study.**
(PDF)
