# Supplementary material for: Hof1 and Rvs167 Have Redundant Roles in Actomyosin Ring Function during Cytokinesis in Budding Yeast
Source: PLoS One. 2013 Feb 28;8(2):e57846. doi: 10.1371/journal.pone.0057846 (PMC3585203; doi:10.1371/journal.pone.0057846)
Supplement: Figure S1 — Interactions between yeast SH3 proteins and regulators of the actin cytoskeleton. SH3 proteins are shown in red, and each black line represents a physical interaction, based on published data summarised in ‘BIOGRID’ (http://thebiogrid.org/). (PDF) [file pone.0057846.s001.pdf]

Nkosi / Targosz Supplementary Figure 1

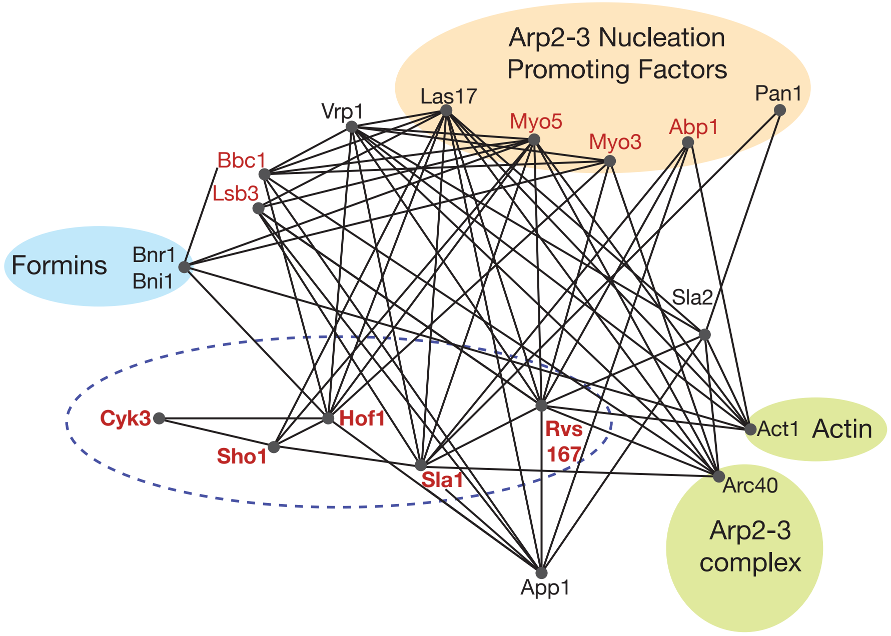

Interactions between yeast SH3 proteins and regulators of the actin cytoskeleton. SH3 proteins are shown in red, and each black line represents a physical interaction, based on published data summarised in 'BIOGRID' (http://thebiogrid.org/).
