# Supplementary material for: Hof1 and Rvs167 Have Redundant Roles in Actomyosin Ring Function during Cytokinesis in Budding Yeast
Source: PLoS One. 2013 Feb 28;8(2):e57846. doi: 10.1371/journal.pone.0057846 (PMC3585203; doi:10.1371/journal.pone.0057846)
Supplement: Figure S2 — The Sho1-Hog1 MAP kinase pathway becomes essential in the absence of Hof1. (A) Tetrad analysis of diploid yeast cells lacking one copy of HOF1 and one copy of SHO1. Spores of the indicated genotypes were grown for 24 hours on YPD plates at 24°C. The scale bars indicate 20 µm. (B) Tetrad analysis of diploid yeast cells lacking one copy of HOF1 and one copy of either HOG1 or PBS2. (C) Spores of the indicated genotypes were grown for 24 hours on YPD plates at 24°C. The scale bars indicate 20 µm. (D) Tetrad analysis of diploid yeast cells lacking one copy of SHO1 and one copy of RVS167. (PDF) [file pone.0057846.s002.pdf]

Nkosi / Targosz Supplementary Figure 2

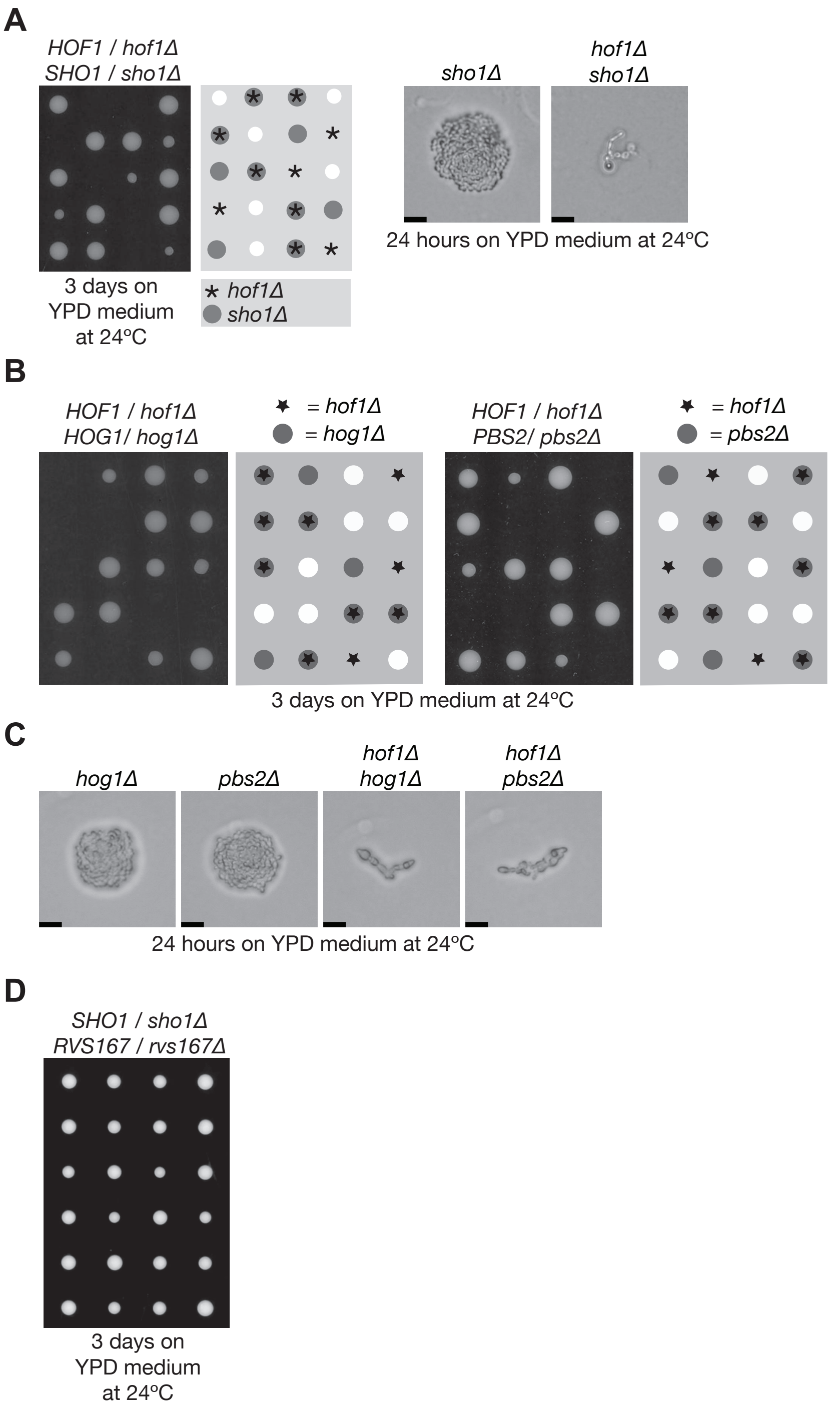

The Sho1-Hog1 MAP kinase pathway becomes essential in the absence of Hof1.
(**A**) Tetrad analysis of diploid yeast cells lacking one copy of *HOF1* and one copy of *SHO1*.
Spores of the indicated genotypes were grown for 24 hours on YPD plates at 24°C.
The scale bars indicate 20μm.
(**B**) Tetrad analysis of diploid yeast cells lacking one copy of *HOF1* and one copy of either *HOG1* or *PBS2*.
(**C**) Spores of the indicated genotypes were grown for 24 hours on YPD plates at 24°C. The scale bars indicate 20μm.
(**D**) Tetrad analysis of diploid yeast cells lacking one copy of *SHO1* and one copy of *RVS167*.
