# Supplementary material for: Hof1 and Rvs167 Have Redundant Roles in Actomyosin Ring Function during Cytokinesis in Budding Yeast
Source: PLoS One. 2013 Feb 28;8(2):e57846. doi: 10.1371/journal.pone.0057846 (PMC3585203; doi:10.1371/journal.pone.0057846)
Supplement: Figure S3 — The rvs167-ΔSH3 allele is not synthetic lethal with either hof1-ΔFBAR or hof1-ΔSH3. (A) Tetrad analysis of diploid yeast cells with one copy of hof1-ΔFBAR and one copy of rvs167-ΔSH3. (B) Tetrad analysis of diploid yeast cells with one copy of hof1-ΔSH3 and one copy of rvs167-ΔSH3. (PDF) [file pone.0057846.s003.pdf]

Nkosi / Targosz Supplementary Figure 3

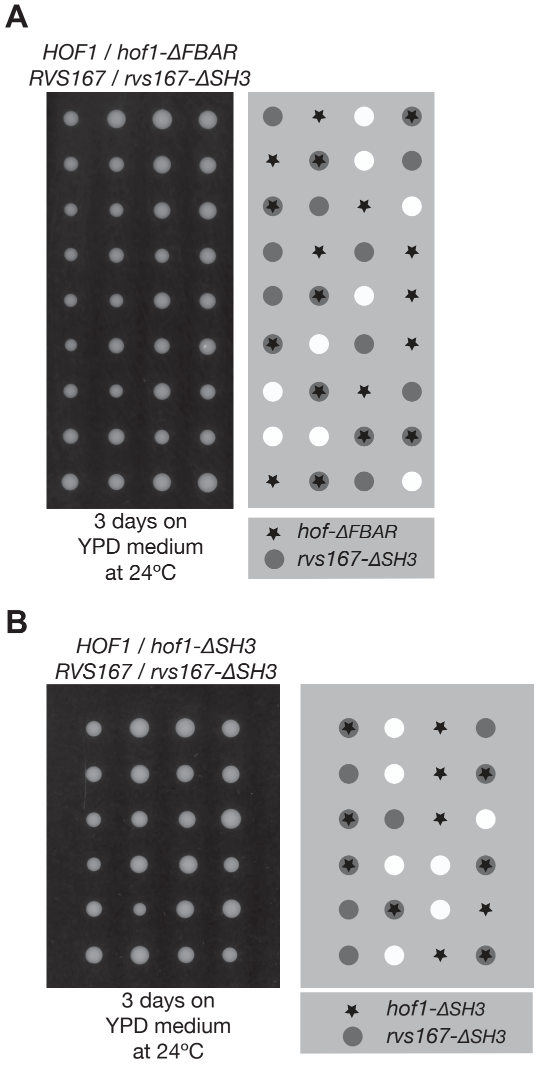

The *rvs167-ΔSH3* allele is not synthetic lethal with either *hof1-ΔFBAR* or *hof1-ΔSH3*.
(**A**) Tetrad analysis of diploid yeast cells with one copy of hof1-ΔFBAR and one copy of rvs167-ΔSH3.
(**B**) Tetrad analysis of diploid yeast cells with one copy of hof1-ΔSH3 and one copy of rvs167-ΔSH3.
