# Supplementary material for: Hof1 and Rvs167 Have Redundant Roles in Actomyosin Ring Function during Cytokinesis in Budding Yeast
Source: PLoS One. 2013 Feb 28;8(2):e57846. doi: 10.1371/journal.pone.0057846 (PMC3585203; doi:10.1371/journal.pone.0057846)
Supplement: Figure S4 — Interaction of the SH3 domain of Rvs167 with Inn1 is specific. (A) In contrast to Rvs167, the SH3 proteins Abp1 and Lsb3 do not interact with Inn1 in the two-hybrid assay. (B) The two-hybrid interaction of Rvs167 with Inn1 is blocked by the Rvs167-P473L mutation in the SH3 domain. (PDF) [file pone.0057846.s004.pdf]

Nkosi / Targosz Supplementary Figure 4

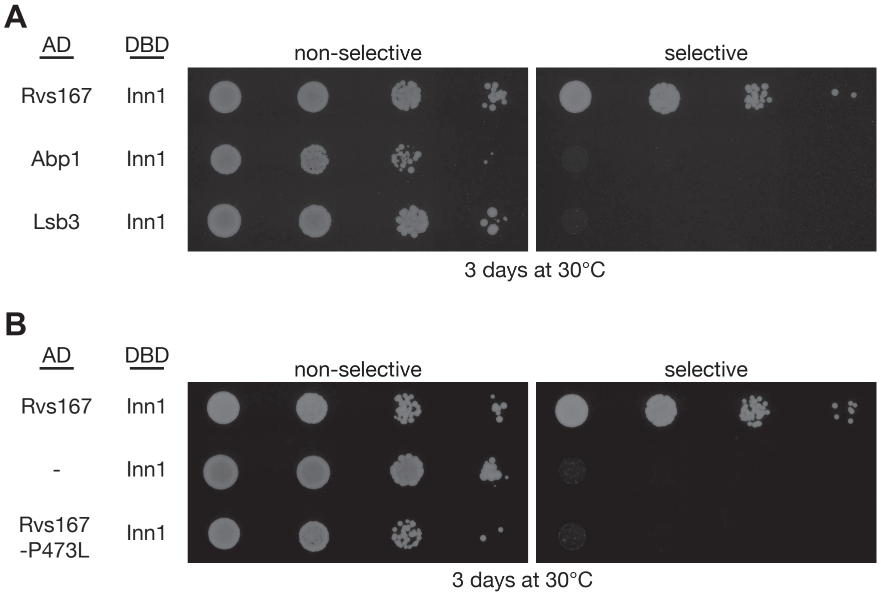

Interaction of the SH3 domain of Rvs167 with Inn1 is specific.
(**A**) In contrast to Rvs167, the SH3 proteins Abp1 and Lsb3 do not interact with Inn1 in the two-hybrid assay.
(**B**) The two-hybrid interaction of Rvs167 with Inn1 is blocked by the Rvs167-P473L mutation in the SH3 domain.
