# Supplementary material for: Hof1 and Rvs167 Have Redundant Roles in Actomyosin Ring Function during Cytokinesis in Budding Yeast
Source: PLoS One. 2013 Feb 28;8(2):e57846. doi: 10.1371/journal.pone.0057846 (PMC3585203; doi:10.1371/journal.pone.0057846)
Supplement: Figure S5 — In contrast to hof1Δ , the rvs167Δ strain grows well at 37°C.Cells were grown for two days on YPD medium at 24°C or 37°C as indicated. (PDF) [file pone.0057846.s005.pdf]

Nkosi / Targosz Supplementary Figure 5

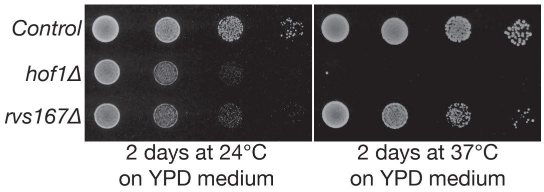

In contrast to *hof1Δ*, the *rvs167Δ* strain grows well at 37°C.
Cells were grown for two days on YPD medium at 24°C or 37°C as indicated.
