# Supplementary material for: Hof1 and Rvs167 Have Redundant Roles in Actomyosin Ring Function during Cytokinesis in Budding Yeast
Source: PLoS One. 2013 Feb 28;8(2):e57846. doi: 10.1371/journal.pone.0057846 (PMC3585203; doi:10.1371/journal.pone.0057846)
Supplement: Table S1 — Yeast strains used in this study. (PDF) [file pone.0057846.s006.pdf]

Supplementary Table I Strains used in this study

Strain	Genotype
W303-1a	*MAT***a** *ade2-1 ura3-1 his3-11,15 trp1-1 leu2-3,112 can1-100*
W303-1b	*MATa ade2-1 ura3-1 his3-11,15 trp1-1 leu2-3,112 can1-100*
W303-1	*MAT***a** *ade2-1 ura3-1 his3-11,15 trp1-1 leu2-3,112 can1-100 / MATa ade2-1 ura3-1 his3-11,15 trp1-1 leu2-3,112 can1-100*
CC1530	*hof1Δ (K.l.TRP1)*
YASD555	*GAL-UBR1 (HIS3) INN1-GFP (kanMX) hof1-td (hphNT)*
YASD720	*GAL-UBR1 (HIS3) INN1-GFP (kanMX)*
YASD810	*GAL-UBR1 (HIS3) INN1-GFP (kanMX) cyk3-td (K.l.TRP1)*
YASD743	*GAL-UBR1 (HIS3) GFP-IQG1 (kanMX)*
YASD832	*HOF1 / hof1Δ (K.l.TRP1) CYK3 / cyk3Δ (hphNT) MAT***a** */ MATα*
YASD864	*GAL-UBR1 (HIS3) INN1-GFP (kanMX) hof1-td (hphNT) cyk3-td (K.l.TRP1)*
YBST58	*HOF1 / hof1Δ (K.l.TRP1) RVS167 / rvs167Δ (hphNT) MAT***a** */ MATα*
YBST62	*HOF1 / hof1Δ (K.l.TRP1) SHO1 / sho1Δ (hphNT) MAT***a** */ MATα*
YBST85	*rvs167Δ (hphNT)*
YBST139	*CYK3 / cyk3Δ (K.l.TRP1) RVS167 / rvs167Δ (hphNT) MAT***a** */ MATα*
YBST194	*GAL-UBR1 (HIS3) INN1-GFP (kanMX) hof1-td (hphNT) rvs167Δ (hphNT)*
YPNK13	*HOF1 / hof1-ΔSH3 (kanMX) RVS167 / rvs167Δ (hphNT) MAT***a** */ MATα*
YPNK16	*HOF1 / hof1Δ (K.l.TRP1) RVS167 / rvs167-ΔSH3 (hphNT) MAT***a** */ MATα*
YPNK27	*GAL-UBR1 (HIS3) GFP-IQG1 (kanMX) hof1-td (hphNT)*
YPNK38	*GAL-UBR1 (HIS3) INN1-GFP (kanMX) hof1-td (hphNT) rvs167-ΔSH3 (hphNT)*
YPNK41	*GAL-UBR1 (HIS3) GFP-IQG1 (kanMX) hof1-td (hphNT) rvs167Δ (hphNT)*
YPNK50	*GAL-UBR1 (HIS3) GFP-IQG1 (kanMX) hof1-td (hphNT) rvs167-ΔSH3 (hphNT)*
YPNK60	*HOF1 / hof1Δ (K.l.TRP1) PBS2 / pbs2Δ (kanMX) MAT***a** */ MATα*

YPNK63	*HOF1 / hof1Δ (K.l.TRP1) HOG1 / hog1Δ (kanMX) MAT***a** */ MATα*
YPNK88	*HOF1 / hof1-ΔSH3 (kanMX) RVS167 / rvs167-ΔSH3 (hphNT) MAT***a** */ MATα*
YPNK150	*GAL-UBR1 (HIS3) INN1-GFP (kanMX) rvs167Δ (hphNT)*
YPNK152	*GAL-UBR1 (HIS3) INN1-GFP (kanMX) hof1-td (hphNT) cyk3-td (K.l.TRP1) rvs167Δ (hphNT)*
YPNK154	*HOF1 / hof1Δ (K.l.TRP1) RVS167 / rvs167-P473L (URA3) MAT***a** */ MATα*
YPNK165	*arp2-2 (URA3) hof1-td (hphNT) INN1-GFP (kanMX) GAL-UBR1 (HIS3)*
YPNK167	*arp2-2 (URA3) INN1-GFP (kanMX) GAL-UBR1 (HIS3)*
YPNK171	*HOF1 / hof1-ΔFBAR (kanMX) RVS167 / rvs167-ΔSH3 (hphNT) MAT***a** */ MATα*
YPNK186	*HOF1 / hof1-ΔFBAR (kanMX) RVS167 / rvs167Δ (hphNT) MAT***a** */ MATα*
YPNK187	*HOF1 / hof1-ΔFBAR (kanMX) CYK3 / cyk3Δ (K.l.TRP1) MAT***a** */ MATα*
YPNK192	*HOF1 / hof1-ΔSH3 (kanMX) CYK3 / cyk3Δ (K.l. TRP1) MAT***a** */ MATα*

Supplementary Table 1: Strains used in this study.

All strains are based on the W303 background (W303-1a and W303-1b haploids, and W303-1 diploid).
